# Comment on Schuderer et al. Risk Factors for Flap Loss: Analysis of Donor and Recipient Vessel Morphology in Patients Undergoing Microvascular Head and Neck Reconstructions. *J. Clin. Med.* 2023, *12*, 5206

**DOI:** 10.3390/jcm13020617

**Published:** 2024-01-22

**Authors:** Ori Bar, Imad Abu El Naaj

**Affiliations:** Department of Oral and Maxillofacial Surgery, Baruch Padeh Medical Center, Faculty of Medicine, Bar Ilan University, Ramat Gan 15208, Israel

We have read with much interest the study by Schuderer et al. [1], which aimed to examine vessel morphology in recipient and donor blood vessels in patients going through head and neck microvascular reconstruction surgeries and to identify predictors for changes in vessels’ diameters in H.E.-stained specimens, thus predicting post-operative flap loss.

The idea to examine changes in blood vessels’ morphology in patients with different common morbidities is an innovative and fascinating one. Such studies may help to build an image of a risk profile in patients that go through these complex surgeries and assist surgeons operating in that field by knowing ahead of which patients are more prone to flap failure and treat and follow these patients with extra care.

However, it is necessary for such reports to provide a clearer explanation regarding the different co-morbidities and diagnoses of the patients in order for other surgeons and readers to make a change in their routine work and take home a more solid message from the study.

The study concluded that the presence of pre-operative radiation was significantly associated with a reduction in the thickness of the recipient vein in the neck yet did not compromise the overall success of the flap. 34% of the patients in the study were exposed to pre-operative radiation, and 63% of the patients were diagnosed with oral squamous cell carcinoma (OSCC), while the stage of the disease was not specified. A few questions are raised regarding these issues- Have most patients diagnosed with OSCC received pre-operative radiation, or was it dependent on the stage of the disease? Which other patients in the research have received pre-operative radiation, and for what reason? Is there a certain protocol or policy in the writer’s institution for pre-operative radiation in OSCC patients? Was post-operative radiation given in these patients as well?

It is important to note that the treatment of pre-operative radiotherapy followed by surgery is an effective modality for many solid tumors, including locally advanced OSCC and squamous cell carcinoma of the head and neck region. Response to radiotherapy is associated with better survival, and the response of the tumor to pre-operative radiotherapy is a strong prognostic factor for treatment outcome [2]. The administration of pre-operative chemotherapy with simultaneous radiotherapy is also described as a possible treatment for these tumors and was shown in the literature to be a practicable and effective method for patients suffering from locally advanced OSCC [3]. Locally advanced oral squamous cell carcinoma stages 3 and 4 are a significant therapeutic challenge, and management of these may require the application of multidisciplinary approaches, as mentioned, combining surgery with post-operative radiation, pre-operative radiochemotherapy, or both [4]. The establishment of neoadjuvant induction radiochemotherapy followed by radical surgery was strengthened in the last several decades, as several reports show this methodology to be practicable with bearable side effects and results in assuring overall survival rates [5,6,7]. The purpose of pre-operative neoadjuvant therapy is to improve local and locoregional control of the diseases, thus allowing a more functional reconstruction after surgery. Different neoadjuvant protocols were established over the years, placing chemoradiotherapy before surgery. An example is a retrospective analysis that showed concurrent radiotherapy with 40 Gy and low-dose chemotherapy followed by surgery can be a practicable and reliable treatment option with encouraging overall and disease-free survival rates [8]. Yet, it was reported that different chemoradiotherapy regimens and administrations do not affect the treatment’s result [9].

While advanced tumors may be treated with these mentioned methods before or after surgery, the first-line treatment for early-stage oral squamous cell carcinoma is either surgery or radiotherapy, and there is an agreement regarding the prognosis with such an approach [5,10,11]. Simultaneous post-operative chemotherapy and radiotherapy significantly improve the rates of local and locoregional control and survival rates in the population of high-risk patients after resection of head and neck cancers. Moreover, it was also reported that the post-operative treatment of concurrent high-dose chemotherapy is even more effective than post-operative radiotherapy alone in patients presenting locally advanced head and neck cancer [12,13].

It should be mentioned that radiotherapy is frequently needed for salivary malignancies, especially in tumors presenting stages T3-T4 and extra parotid invasions [14], and that radiotherapy has a place in the definitive or adjuvant management of skin cancers [15]. A number of the patients in the study suffered from these pathologies [1], and more light should be shed on whether radiotherapy was given to these patients as well.

Studies such as that of Schuderer et al. [1] have a meaningful purpose and must continue to be conducted. Understanding the way common co-morbidities affect the microvascular free flap is crucial for the correct and successful management of patients, both pre and perioperatively. Yet, it is important to understand a major unaddressed aspect in this study, that of how so many patients received pre-operative radiation and for what reason or indication.

Subsequent research endeavors in that field should address the topic of pre-operative radiation more informatively while discussing the considerations behind giving it. Sharing the institution’s treatment protocol for different pathologies may provide additional information and clarify the decision-making behind the presented results.

## References

[1] Schuderer J.G., Dinh H.T., Spoerl S., Taxis J., Fiedler M., Gottsauner J.M., Maurer M., Reichert T.E., Meier J.K., Weber F. (2023). Risk Factors for Flap Loss: Analysis of Donor and Recipient Vessel Morphology in Patients Undergoing Microvascular Head and Neck Reconstructions. J. Clin. Med..

[2] Eder-Czembirek C., Czembirek C., Selzer E. (2016). Neoadjuvant radiotherapy plus radical surgery for locally advanced stage III/IV oral cancer: Analysis of prognostic factors affecting overall survival. Oral Oncol..

[3] Kawano S., Zheng Y., Oobu K., Matsubara R., Goto Y., Chikui T., Yoshitake T., Kiyoshima T., Jinno T., Maruse Y. (2016). Clinicopathological evaluation of pre-operative chemoradiotherapy with S-1 as a treatment for locally advanced oral squamous cell carcinoma. Oncol. Lett..

[4] Shingaki S., Takada M., Sasai K., Bibi R., Kobayashi T., Nomura T., Saito C. (2003). Impact of lymph node metastasis on the pattern of failure and survival in oral carcinomas. Am. J. Surg..

[5] Kirita T., Ohgi K., Shimooka H., Yamanaka Y., Tatebayashi S., Yamamoto K., Mishima K., Sugimura M. (1999). Preoperative concurrent chemoradiotherapy plus radical surgery for advanced squamous cell carcinoma of the oral cavity: An analysis of long-term results. Oral Oncol..

[6] Eckardt A., Wildfang I., Karstens J.H. (1999). Simultane Radiochemotherapie mit Taxol/Carboplatin bei fortgeschrittenen operablen Kopf-Hals-Tumoren. Vorläufige Ergebnisse [Simultaneous radiochemotherapy with taxol/carboplatin in advanced operable head-neck tumors. Preliminary results]. Strahlenther. Onkol..

[7] Slotman G.J., Doolittle C.H., Glicksman A.S. (1992). Preoperative combined chemotherapy and radiation therapy plus radical surgery in advanced head and neck cancer. Five-year results with impressive complete response rates and high survival. Cancer.

[8] Freier K., Engel M., Lindel K., Flechtenmacher C., Mühling J., Hassfeld S., Hofele C. (2008). Neoadjuvant concurrent radiochemotherapy followed by surgery in advanced oral squamous cell carcinoma (OSCC): A retrospective analysis of 207 patients. Oral Oncol..

[9] Yamakawa N., Harada H., Nakayama H., Ohiro Y., Bukawa H., Kirita T. (2024). Preoperative chemoradiotherapy for locally advanced oral squamous cell carcinoma: A multicenter retrospective study. J. Oral Maxillofac. Surg. Med. Pathol..

[10] Mücke T., Konen M., Wagenpfeil S., Kesting M.R., Wolff K.D., Hölzle F. (2011). Low-dose preoperative chemoradiation therapy compared with surgery alone with or without postoperative radiotherapy in patients with head and neck carcinoma. Ann. Surg. Oncol..

[11] Klug C., Berzaczy D., Voracek M., Millesi W. (2008). Preoperative chemoradiotherapy in the management of oral cancer: A review. J. Craniomaxillofac. Surg..

[12] Cooper J.S., Pajak T.F., Forastiere A.A., Jacobs J., Campbell B.H., Saxman S.B., Kish J.A., Kim H.E., Cmelak A.J., Rotman M. (2004). Postoperative concurrent radiotherapy and chemotherapy for high-risk squamous-cell carcinoma of the head and neck. N. Engl. J. Med..

[13] Bernier J., Domenge C., Ozsahin M., Matuszewska K., Lefèbvre J.L., Greiner R.H., Giralt J., Maingon P., Rolland F., Bolla M. (2004). Postoperative irradiation with or without concomitant chemotherapy for locally advanced head and neck cancer. N. Engl. J. Med..

[14] Larnaudie A., Marcy P.Y., Delaby N., Costes Martineau V., Troussier I., Bensadoun R.J., Vergez S., Servagi Vernat S., Thariat J. (2022). Radiotherapy of salivary gland tumours. Cancer Radiother..

[15] Mierzwa M.L. (2019). Radiotherapy for Skin Cancers of the Face, Head, and Neck. Facial. Plast. Surg. Clin. N. Am..

